# Fish isoallergens and variants: database compilation, *in silico* allergenicity prediction challenges, and epitope-based threshold optimization

**DOI:** 10.3389/fbinf.2025.1669237

**Published:** 2025-10-20

**Authors:** Vachiranee Limviphuvadh, Thimo Ruethers, Minh N. Nguyen, Dean R. Jerry, Benjamin P. C. Smith, Yulan Wang, Yansong Miao, Anand Kumar Andiappan, Andreas L. Lopata, Sebastian Maurer-Stroh

**Affiliations:** 1 Biomolecular Sequence To Function Division (BSFD), Bioinformatics Institute (BII), Agency for Science, Technology and Research (A*STAR), Singapore, Singapore; 2 Tropical Futures Institute, James Cook University, Singapore, Singapore; 3 Centre for Sustainable Tropical Fisheries and Aquaculture, College of Science and Engineering, James Cook University, Townsville, QLD, Australia; 4 Molecular Allergy Research Laboratory, College of Science and Engineering, James Cook University, Townsville, QLD, Australia; 5 Biomolecular Structure To Mechanism Division (BSMD), Bioinformatics Institute (BII), Agency for Science, Technology and Research (A*STAR), Singapore, Singapore; 6 Monell Chemical Senses Center, Philadelphia, PA, United States; 7 Singapore Phenome Center, Lee Kong Chian School of Medicine, Nanyang Technological University, Singapore, Singapore; 8 School of Biological Sciences, Nanyang Technological University, Singapore, Singapore; 9 Singapore Immunology Network (SIgN), Agency for Science, Technology and Research (A*STAR), Singapore, Singapore; 10 Yong Loo Lin School of Medicine and Department of Biological Sciences, National University of Singapore (NUS), Singapore, Singapore

**Keywords:** fish allergy, isoallergens and variants, food allergens, parvalbumin, B-cell epitope mapping, phylogenetic tree, protein allergenicity prediction, *in silico* prediction tools

## Abstract

**Introduction:**

Fish is a major food allergy trigger with a complex variety of allergenic protein isoforms and vast species diversity exhibiting variable allergenicity. This is the first study to systematically compile fish isoallergen and variant entries associated with ingestion-related allergic reactions.

**Methods:**

Entries were compiled from four major allergen databases: World Health Organization and International Union of Immunological Societies (WHO/IUIS), AllergenOnline, Comprehensive Protein Allergen Resource (COMPARE), and Allergome, including evidence from *in vitro* IgE-binding assays and complete amino acid sequences. Challenges in predicting the allergenicity of fish isoallergens and variants were evaluated, and the sensitivity of five widely used *in silico* tools (AllerCatPro 2.0, AlgPred 2.0, pLM4Alg, AllergenFP v.1.0, and AllerTop v.2.0) was assessed. Epitope mapping and phylogenetic analyses were performed for the major fish allergen parvalbumin, incorporating experimentally validated B-cell epitope data from the Immune Epitope Database (IEDB) and evolutionary relationships.

**Results:**

A comprehensive dataset of 79 unique fish isoallergen and variant entries from 34 fish species was identified, with 25 entries common across all four databases. AllerCatPro 2.0 achieved the highest sensitivity (97.5%). A phylogenetic tree was constructed, integrating epitope data to optimize protein family-specific thresholds for differentiating allergenic from less/non-allergenic parvalbumins. A threshold of ≥4 IEDB-mapped epitopes allowing up to two mismatches captured 52 out of 54 parvalbumin sequences (96%) in the dataset, effectively distinguishing between parvalbumin classes.

**Discussion:**

This study enhances understanding of fish allergy by systematically compiling fish isoallergens and variants and integrating B-cell epitope data. The optimized thresholds improve the performance of allergenicity prediction tools and can be applied to other protein families in future studies.

## 1 Introduction

Fish allergy is an increasing public health concern, with reported global prevalence ranging from 0% to 7%, and is associated with a high risk of anaphylaxis and lifelong persistence (Moonesinghe et al., 2016; Conrado et al., 2021). Reliable diagnosis and management are hindered by a lack of tools to assess region-, species- and preparation-specific risks of allergic reactions (Ruethers et al., 2019; Ruethers et al., 2020; Davis et al., 2020).

Predicting the allergenicity of a fish species presents major challenges due to species diversity and complex under-investigated protein repertoires with varying allergenic potentials (Ruethers et al., 2018b). Eleven fish allergens have been identified to date of which parvalbumin (PV) is the best investigated (Dramburg et al., 2023). The allergenic properties of fish proteins vary widely between species and even among isoforms within the same species (Kalic et al., 2021), highlighting the critical importance of focusing on isoallergens and variants. For instance, seven distinct PV isoforms were described in Indian mackerel while 21 PV genes were found in the carp genome (Ruethers et al., 2018a; Mukherjee et al., 2021). Notably, a unique epitope associated with salmon PV (beta-1) isoallergen has been identified as an allergy marker for salmonoid mono-sensitization (Perez-Gordo et al., 2011; Kuehn et al., 2014). In contrast to β-PVs (PVB), α*-PV*s (PVA) are less allergenic and predominantly expressed in cartilaginous fish (Stephen et al., 2017; Kalic et al., 2019; O’Malley et al., 2024). The amino acid sequence of thousands of potential fish allergens is known (www.ncbi.nlm.nih.gov/protein/) of which only a few have been evaluated for their allergenic potential *in vitro* or *in silico*. Species- and isoform-specific allergenic potentials are evident, underscoring the complexity of fish allergies and highlighting the need for further research.

The World Health Organization and International Union of Immunological Societies (WHO/IUIS) Allergen Nomenclature system classifies isoallergens as homologous allergens from the same species with at least 67% sequence identity, similar molecular size, and identical or similar biological function, denoted by two digits after the allergen number (e.g., Clu h 1.01, Clu h 1.02). Isoform variants, which share >90% sequence identity, are identified by two additional digits (e.g., Gad m 1.0101, Gad m 1.0102). This classification system enhances the precise identification of allergenic proteins, aiding research, clinical diagnosis, and regulatory assessments (Pomés et al., 2018). In the present study, we focus on isoallergens and their variants as defined above, considering only those associated with ingestion-related allergic reactions and supported by evidence from *in vitro* IgE-binding assays, in order to distinguish them from non-allergenic isoforms. Understanding this distinction is crucial for interpreting allergenicity data and guiding the systematic compilation and analysis of fish isoallergens in our work.

*In silico* tools for allergen prediction represent a critical advancement in protein assessments, employing computational methods to enhance the identification and evaluation of potential allergens based on protein sequences and structures. This emerging field plays a vital role in promoting food safety and public health, particularly as the prevalence of food allergies continues to rise globally, especially in developed countries where increasing urbanization and lifestyle changes have been associated with this trend (Sampath et al., 2021). Tools such as AllerCatPro 2.0 (Nguyen et al., 2022), AlgPred 2.0 (Sharma et al., 2021), pLM4Alg (Du et al., 2024), AllergenFP v1.0 (Dimitrov et al., 2014b), and AllerTop v2.0 (Dimitrov et al., 2014a) provide powerful resources for predicting allergenicity, employing diverse methodologies with varying degrees of accuracy. These tools complement expensive laboratory experimentation by offering high-throughput computational approaches. AllerCatPro 2.0 achieves high prediction accuracy by analyzing both amino acid sequences and three-dimensional structures, utilizing an extensive database of known allergens (Nguyen et al., 2022). AlgPred 2.0 employs a hybrid approach that integrates multiple machine learning techniques to predict allergenic and non-allergenic proteins based on a comprehensive dataset (Sharma et al., 2021). pLM4Alg utilizes pretrained protein language models to analyze protein sequences, extracting meaningful features that are processed through convolutional neural networks to identify patterns (Du et al., 2024). In contrast, AllergenFP v1.0 adopts a fingerprinting method that captures the physiochemical properties of proteins, enabling effective classification between allergens and non-allergens (Dimitrov et al., 2014b). AllerTop v2.0 focuses on the physiochemical properties of proteins, utilizing z-descriptors and auto-cross covariance transformations to represent protein sequences uniformly, thus improving prediction accuracy significantly (Dimitrov et al., 2014a). Each tool contributes unique features to the field of allergen prediction, offering insights into the strengths and limitations of *in silico* methodologies. Despite their advancements, these tools face challenges such as false positives, false negatives, and reduced reliability in predicting which food proteins are likely to cause new food allergies, a phenomenon known as *de novo* sensitization.

We aimed to consolidate and comprehensively evaluate the knowledge about fish isoallergens and variants by conducting a systematic review of four widely used and frequently updated allergen databases -WHO/IUIS (Pomés et al., 2018), AllergenOnline (Goodman et al., 2016), Comprehensive Protein Allergen Resource (COMPARE) (van Ree et al., 2021) and Allergome (Mari et al., 2009). To our knowledge, this is the first study to systematically compile fish food isoallergens and their variants. Subsequently, we assessed the allergenicity prediction sensitivity of the five above-mentioned widely used *in silico* tools. Finally, we highlight how applying a protein family-specific threshold, combined with epitope data and AllerCatPro 2.0 results, enhances the performance of allergenicity predictions.

## 2 Materials and methods

### 2.1 Collating and compiling fish isoallergens from four allergen databases

All fish allergens were collated from the four major allergen databases: WHO/IUIS, AllergenOnline, COMPARE and Allergome. Details collected from each database included isoallergens and their variants (if available), allergen name, corresponding accession IDs (UniProt, RefSeq, GenBank), original biochemical names, PMID (PubMed Identifier) and species for each entry. Selection criteria focused on fish isoallergens implicated in allergic reactions through ingestion (i.e., food allergens), supported by evidence from *in vitro* IgE-binding assays (e.g., IgE immunoblotting) that demonstrate IgE recognition. Only fish allergens with complete amino acid sequences were considered for further analysis. UniProt IDs were prioritized over other accession IDs. When UniProt IDs were not available, RefSeq or GenBank IDs were used as alternatives. For clarity, the access dates of all databases used in this study are summarized here: WHO/IUIS (16 November 2022), AllergenOnline (16 November 2022), COMPARE (6 February 2023), and Allergome (15 March 2023). These dates are also provided in the respective database subsections. The steps for collecting fish allergens from each database were as follows:

#### 2.1.1 WHO/IUIS

The “Tree View” tab of the WHO/IUIS database (https://allergen.org/) was accessed on 16 November 2022. The search was narrowed to “*Animalia Chordata”*, and further restricted to “food allergens”. Fish species were then manually selected and collected. WHO/IUIS database information was prioritized as it contains the most extensively reviewed fish isoallergens and their variants, and entries from other databases were manually annotated to ensure consistency.

#### 2.1.2 AllergenOnline

AllergenOnline v.21 (Goodman et al., 2016), released on 14 February 2021 (http://www.allergenonline.org/databasebrowse.shtml), was reviewed on 16 November 2022. The search was refined to “Type: Food Animal” and the data were downloaded. Subsequently, only fish species were manually selected. Some entries were annotated with IUIS-assigned isoallergens and variants, while others were manually annotated using provided accession IDs to identify any corresponding IUIS-assigned isoallergens and variants. If no matches were found, the entries were retained as accession IDs in the dataset.

#### 2.1.3 COMPARE

The COMPARE database 2023, released on 01/26/2023 (http://db.comparedatabase.org/) was accessed on 6 February 2023, containing 2,631 entries. The data were downloaded, and fish allergens were manually collected. Since COMPARE provides only the IUIS-assigned allergen names (e.g., Gad m 1), entries were manually annotated by matching them with corresponding IUIS-assigned isoallergens and variants using accession IDs. Entries without matches were retained as accession IDs in the dataset.

#### 2.1.4 Allergome

The Allergome database (https://www.allergome.org/) was accessed on 15 March 2023, containing 7,535 entries. From the “Allergens” tab, the “advanced search” page was accessed. The following criteria were applied: Allergens: *Molecule*; Molecule Option: *tick off “No Isoform and Epitopes”*; Routes of Exposure: *Ingestion*; Sources: *fish*. Additionally, in the “plus Search for Molecule Scoring” section, Data Generation: *Experimental from Literature*; Sequence: *Available*; IgE Non-Functional Test: *Positive* were selected. For Allergome, only IUIS-assigned isoallergen and variant entries were manually selected, while other entries were excluded to avoid duplication.

Once amino acid sequences in FASTA format were retrieved for each of the unique entries compiled from the four databases, pairwise %identity was calculated for each allergenic protein group using Clustal Omega (version 2.1) with ClustalW parameters (Madeira et al., 2024). The resulting %identity matrices were processed in Excel, where self-comparisons and values of 100% were excluded, and the mean %identity ±SD (min–max) was calculated across the remaining pairwise values. For groups with only two sequences, the single pairwise %identity was reported without SD.

### 2.2 Evaluating the allergenicity prediction sensitivity of *in silico* tools

Amino acid sequences in FASTA format were retrieved for each of the 79 accession IDs corresponding to unique fish isoallergens and variants compiled from the four databases as detailed in Section 2.1. Allergenicity prediction were retrieved using the following tools: AllerCatPro 2.0 (https://allercatpro.bii.a-star.edu.sg/), AlgPred 2.0 (https://webs.iiitd.edu.in/raghava/algpred2/index.html), pLM4Alg (https://f6wxpfd3sh.us-east-1.awsapprunner.com/), AllergenFP v.1.0 (https://ddg-pharmfac.net/AllergenFP/), and AllerTop v.2.0 (https://www.ddg-pharmfac.net/allertop_test/), corresponding to the five most widely used tools.

For AllerCatPro 2.0, the 79 sequences were submitted to obtain results in a comma-separated table format. For AlgPred 2.0, the sequences were submitted using the “Prediction of Allergens” batch tool (https://webs.iiitd.edu.in/raghava/algpred2/batch.html) with the default Hybrid option (RF + BLAST + MERCI for machine learning technique and a threshold of 0.3) selected. Predictions of allergen or non-allergen were recorded along with detailed results. For pLM4Alg, the pLM4Alg-640 model was selected, and protein sequences were input to retrieve allergen or non-allergen predictions for each sequence. The number following “pLM4Alg” (e.g., pLM4Alg-640) represents the dimensionality of the output embeddings used by the model to analyze protein sequences (Du et al., 2024). The pLM4Alg-640 model was chosen as it provides the highest-dimensional model available on the website. For AllergenFP v.1.0, amino acid sequences were submitted to retrieve predictions, including classifications as either probable allergen or probable non-allergen, the highest Tanimoto similarity index, and the protein with the best-hit allergens. Similarly, for AllerTop v.2.0, sequences were submitted to obtain predictions (either probable allergen or probable non-allergen) and the nearest protein predicted by the tool. Since AlgPred 2.0, AllergenFP v.1.0, and AllerTOP v.2.0 cannot process sequences with absent or nonstandard amino acids, any sequence encountering this issue was classified as a non-allergen when calculating the % sensitivity of the predictions.

### 2.3 Epitope identification and phylogenetic analysis of parvalbumins

Fish allergen epitopes were accessed and searched in the Immune Epitope Database (IEDB, www.iedb.org) on 10 April 2023. Search parameters included: Epitope: Any; Host: Human; Disease: Allergic; Assay: T cell, B cell, MHC Ligand, with separate searches for Positive and Negative outcomes. For the Epitope Source, we selected Fish by browsing the taxonomy tree under Eukaryotes > Metazoan > Vertebrates > Fish. The search results were exported as an Excel file. In this study, IEDB epitopes identified from assays with positive outcomes are referred to as “positive IEDB epitopes”, while those with negative outcomes are referred to as “negative IEDB epitopes”. For further analysis, we included only linear PV epitopes.

A phylogenetic tree was constructed using seven representative bony fish PVs from our compiled data: Gad m 1.0101, Sal s 1.0101, Cyp c 1.0101, Sco s 1.0101, Pan h 1.0101, Lat c 1.0101 and Lat c 1.0201. Additionally, PV from mangrove red snapper (UniProt: A5YVT7), PVB from Megrim (Lep w 1.0101, UniProt: B5WX08), two PVs from cartilaginous fish (*Spotless smooth-hound SPV-I*, PDB: 5ZGM_A; and *Thornback ray PVA,* UniProt: P02630), two PVs from edible frogs (Ran e 1.0101, PVA, UniProt: Q8JIU2; Ran e 2.0101, PVB, UniProt: Q8JIU1), two PVs from saltwater crocodile (Cro p 1.0101, PVB, UniProt: A0A7M4EAX1; Cro p 2.0101, PVA, RefSeq: XP_019400389) as well as PVs from human (UniProt: P20472), cow (UniProt: Q0VCG3), pig (UniProt: A0A287ALJ2), and chicken (UniProt: C1L370) were included as representative non-fish vertebrates. Finally, another type of parvalbumin, oncomodulin, from human (UniProt: P0CE72) was also included. These 20 sequences were compiled into a FASTA file and aligned using MAFFT version 7 (https://mafft.cbrc.jp/alignment/server/) (Katoh et al., 2019) with default parameters. The aligned sequences were used to construct a phylogenetic tree using the Maximum Likelihood method based on the JTT matrix-based model (Jones et al., 1992) with a Gamma distribution to model evolutionary rate differences among sites. Bootstrap analysis was performed with 500 replicates. The tree was drawn to scale, with branch lengths measured in substitutions per site. All evolutionary analyses were conducted in MEGA X (Kumar et al., 2018).

After constructing the phylogenetic tree, six data points were mapped onto the tree: %identity linear 80 aa window, %identity 3D epitope, #positive IEDB, #positive IEDB-2M, #negative IEDB, and #negative IEDB-2M. The first two data points were retrieved from AllerCatPro 2.0 results. The remaining four were calculated for each sequences: the number of positive IEDB epitopes mapped to the sequence (#positive IEDB), positive IEDB epitopes mapped with up to two mismatches allowed (#positive IEDB-2M), negative IEDB epitopes mapped to the sequence (#negative IEDB), and negative IEDB epitopes mapped with up to two mismatches allowed (#negative-2M).

**FIGURE 1:**
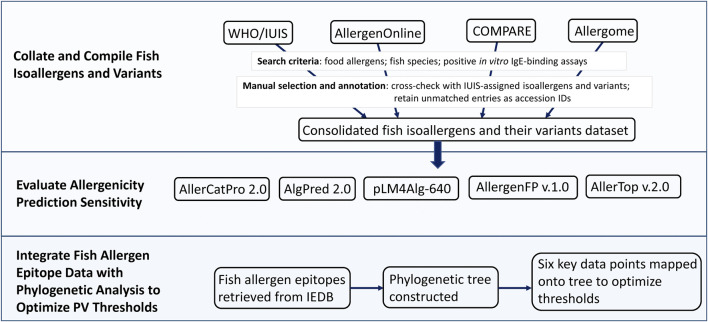
Workflow of the study. The workflow is summarized in three steps: (1) Collate and compile fish isoallergens and variants from four major allergen databases, (2) Evaluate allergenicity prediction sensitivity using five *in silico* prediction tools, and (3) Integrate fish allergen epitope data with phylogenetic analysis to optimize PV (parvalbumin) thresholds.

Figure 1 shows a simple workflow summarizing the main steps of the study.

## 3 Results and discussion

### 3.1 Seventy-nine unique fish isoallergen and variant entries with complete amino acid sequences

From the WHO/IUIS database, 109 allergens were identified in *Animalia Chordata* out of a total of 1,089 total entries. When restricted to food allergens, this number was reduced to 67. Of these, 41 IUIS-assigned allergen names, comprising 57 IUIS-assigned isoallergens and variants across 19 fish species, were manually curated by selecting only fish species (Table 1; Supplementary Figure S1A). WHO/IUIS database information was prioritized due to its extensive review of isoallergens and variants, including positive experimental data on specific IgE binding with at least five sera from patients allergic to the respective allergen source (Pomés et al., 2018). This information was used as a reference when collecting data from other databases.

**TABLE 1:** Summary of fish IUIS-assigned, unassigned, and complete sequence entries per database (number of entries).

Database	IUIS-assigned isoallergens and variants	Unassigned IUIS entries	Subtotal	Complete sequences for analysis
WHO/IUIS	57	—	57	50
AllergenOnline	54	32	86	73
COMPARE	33	49	82	56
Allergome	55	—	55	49
Total (unique fish isoallergens and variants)			**107**	**79**

Subtotal refers to the sum of IUIS-assigned and unassigned entries for each database. The last row shows the total number of unique fish isoallergens and variants and the number of complete sequences used for downstream analysis.

In the AllergenOnline database, 259 of 2,233 entries were classified as “Type: Food Animal”, indicating food allergens derived from animals. Of these, 92 entries originated from fish species. These comprised 65 entries categorized as “IgE but no biological test” and 27 as “IgE plus basophil + or SPT+”. After annotating entries with IUIS-assigned isoallergens and variants using accession IDs and removing duplicate isoallergen and variant entries, such as Sal s 1.0101, which had three entries due to different sequence IDs, 86 unique entries remained. This included 54 IUIS-assigned isoallergens and variants from 19 species and 32 unassigned IUIS entries, all identified as PVB from 17 species (Table 1; Supplementary Figure S1B).

From the COMPARE database, 89 of 2,631 entries were identified as fish-derived allergens through manual selection. Each entry was then annotated by matching it with the corresponding IUIS-assigned isoallergen and variant using the provided accession IDs, as COMPARE lists only the IUIS-assigned allergen name if available. Following the removal of duplicate isoallergen and variant entries, 33 IUIS-assigned isoallergens and variants from 15 species and 49 unassigned IUIS entries from 21 species were identified (Table 1; Supplementary Figure S1C).

From the Allergome database, 444 of 7,535 entries were identified through advanced search filtering using the criteria: Allergens: *Molecule*, Molecule Option: *tick off “No Isoform and Epitopes”*, Routes of Exposure: *Ingestion*, Sources: *fish*. This number was further reduced to 180 entries by applying an additional filter for positive IgE non-functional tests. Allergome treats single allergen names and isoallergens and variants as distinct entries; for example, Clu h 1 (https://www.allergome.org/script/dettaglio.php?id_molecule=2827) and Clu h 1.0101 (https://www.allergome.org/script/dettaglio.php?id_molecule=6101) are listed as separate entries despite referring to the same allergen. To avoid duplication, only the IUIS-assigned isoallergen and variant entries were retained and 39 entries with allergen names only were excluded. Additionally, 86 unassigned IUIS allergen name entries were excluded since this study focused on isoallergens and variants. Ultimately, 55 isoallergens and variants were curated from Allergome (Table 1; Supplementary Figure S1D).

In total, 107 unique fish isoallergens and variants were collected (Table 1). Table 1 summarizes key metrics for each database, including the number of IUIS-assigned isoallergens and variants, unassigned IUIS entries, and complete sequences used for downstream analysis. Detailed information for each is provided in Supplemantary Table 1, which includes the following: isoallergen and variant names, allergen name, common biochemical name, species, common species name, gene entry URL, GenBank Nucleotide ID, GenBank Protein ID (and/or other accessions excluding Uniprot ID), UniProt ID. Additional information includes amino acid length, sequence completeness (complete, partial, or absent), original biochemical name, PubMed ID (PMID), availability of the entry in relevant databases, and the corresponding FASTA sequence. The second sheet of Supplementary Table S1 provides data on the 57 unique references cited for these entries across the four databases. Of the 107 entries, 79 (approximately 74%) contained complete amino acid sequences, 26 had partial sequences, and two lacked sequence data entirely. Among the 26 partial sequences, five (Gad m 2.0101, Gad m 3.0101, Onc k 5.0101, Thu a 2.0101, and Thu a 3.0101) were found in all four databases. During data collection (November 2022 – March 2023), sequences for Lat c 6.0301 and Sole s 1.01 were unavailable. The RefSeq entry for Lat c 6.0301 (XP_018522130.1) listed in WHO/IUIS (https://allergen.org/viewallergen.php?aid=955) was found to be obsolete. Meanwhile, a later update in December 2024 showed that Sole s 1.01 (https://allergen.org/viewallergen.php?aid=1071) now includes sequences in UniProt (A0A8J9X0D3) and GenBank Protein (CAG9039724). However, since these sequences were unavailable at the time of our data collation, Sole s 1.01 was not included in the presented analysis. Ultimately, the 79 entries with complete sequences were used for further analysis.

**FIGURE 2:**
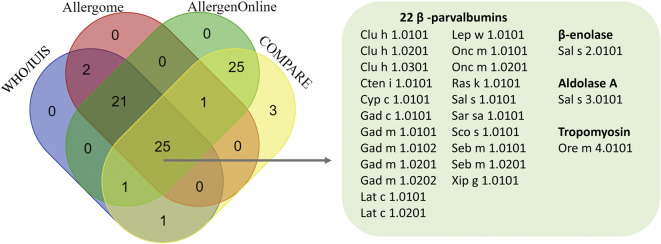
Venn diagram illustrating the number of fish isoallergens identified across four databases, highlighting 25 common isoallergens and their variants. Venn diagram created using https://bioinformatics.psb.ugent.be/webtools/Venn/.

A Venn diagram of the 79 unique fish isoallergens and variants from the four databases revealed that 25 were common to all four databases (Figure 2). Among these, 22 were identified as PVB from 13 fish species: Atlantic herring (Clu h 1.0101, Clu h 1.0201, Clu h 1.0301), Grass carp (Cten i 1.0101), Common carp (Cyp c 1.0101), Baltic cod (Gad c 1.0101), Atlantic cod (Gad m 1.0101, Gad m 1.0102, Gad m 1.0201, Gad m 1.0202), Barramundi (Lat c 1.0101, Lat c 1.0201), Megrim (Lep w 1.0101), Rainbow trout (Onc m 1.0101, Onc m 1.0201), Indian mackerel (Ras k 1.0101), Atlantic salmon (Sal s 1.0101), Pacific pilchard (Sar sa 1.0101), Atlantic mackerel (Sco s 1.0101), and Ocean perch (Seb m 1.0101, Seb m 1.0201). Other common entries included β-enolase and aldolase A from Atlantic salmon (Sal s 2.0101 and Sal s 3.0101, respectively), and tropomyosin from Mozambique tilapia (Ore m 4.0101).

Another 25 allergens were exclusively identified in AllergenOnline and COMPARE but not in WHO/IUIS and Allergome. All of these isoallergens were PVB derived from 16 fish species, including Southern hake, Silver hake, Brook trout, Japanese horse mackerel and Chub mackerel (Figure 2; Supplementary Table S1). In total, we identified 11 common biochemical names for 79 unique known fish entries originating from 34 fish species, compiled from the four databases (Table 2). These biochemical names include PVB, collagen α, tropomyosin, β-enolase, aldolase, creatine kinase, pyruvate kinase, triophosphate isomerase (TPI), glucose 6-phosphate dehydrogenase (GAPDH), L-lactate dehydrogenase (LDH) – altogether referred to as the 11 fish allergens. Among these, PVB was the most prevalent, with 54 entries from 33 fish species, followed by seven collagen α entries from three fish species and four tropomyosin entries from three fish species (Table 2; Supplementary Table S1). Among the 54 PVB entries, half were IUIS-assigned isoallergens and variants, while the remaining half were unassigned. For Collagen α, six out of seven entries were IUIS-assigned, and all other allergenic protein groups were IUIS-assigned (Table 2). PVB entries showed the greatest sequence variability, with a mean pairwise sequence identity (%Identity ±SD, min-max) of 70.81 ± 9.97 (41.5–99.1), ranging from 41.5% (Onc m 1.0201 vs. SPV-I) to 99.1% (Gad m 1.0201 vs. Gad m 1.0202). Notably, some IUIS-assigned isoallergens and variants shared as little as 57% identity (Pan h 1.0101 vs. Pan h 1.0201). Onc m 1.0101 and Onc m 1.0201 shared 64% identity, yet Onc m 1.0101 exhibited 86% identity with PV from Brook trout (GenBank: CAX32966.1). Collagen α entries also displayed variability (72.56 ± 15.14, 59.8–98.1). These results illustrate substantial sequence diversity among isoallergens and variants, even within the same family and species, highlighting the complexity of fish allergens. This variability underscores the need to consider both isoform and species diversity when evaluating allergenic potential and shows that isoallergens of a parent protein may share higher identity with homologs from other species than with isoforms from the same species.

**TABLE 2:** List of 11 common biochemical names for 79 isoallergens and their variants identified across 34 fish species from four databases, with mean %identity ±SD (min–max) for each allergenic protein group.

Common biochemical name	Total entries (IUIS-assigned isoallergens and variants; unassigned IUIS entries)	Number of fish species found	Mean %identity ±SD (min-max)^a^
β-parvalbumin (PVB)	54 (27; 27)	33	70.81 ± 9.97 (41.5–99.1)
Collagen α	7 (6; 1)	3	72.56 ± 15.14 (59.8–98.1)
Tropomyosin	4 (4; 0)	3	88.15 ± 6.6 (80.3–95.4)
β-enolase	3 (3; 0)	3	92.31 ± 1.45 (90.6–93.8)
Aldolase A	2 (2; 0)	2	87.6
Creatine kinase	2 (2; 0)	2	87.37
Triosephosphate isomerase (TPI)	2 (2; 0)	2	85.43
Pyruvate kinase	2 (2; 0)	2	85.66
L-lactate dehydrogenase (LDH)	1 (1; 0)	1	—
Glucose 6-phosphate isomerase (GPI)	1 (1; 0)	1	—
Glyceraldehyde-3-phosphate dehydrogenase (GAPDH)	1 (1; 0)	1	—
Total	79	34	—

^a^ Mean %identity ±SD (min–max) is reported only for allergenic protein groups with three or more sequences; for groups with only two sequences, the mean %identity is the single pairwise value.

### 3.2 Limited allergenicity prediction sensitivity of *in silico* tools

Complete amino acid sequences were available for the identified 79 unique fish allergens. The sensitivity of five *in silico* tools was assessed using these allergens. Here, sensitivity refers to the proportion of true positives correctly predicted by the tools. This evaluation does not account for whether the predicted allergens correspond exactly to the input allergens, as tools like AlgPred 2.0 and pLM4Alg are limited to binary classification models, that is, allergens or non-allergens.

**FIGURE 3:**
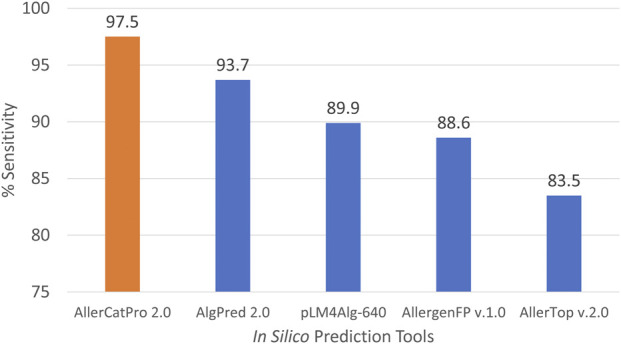
Prediction sensitivity across all identified fish isoallergens (n = 79) for five different *in silico* prediction tools.

Additionally, AlgPred 2.0, AllergenFP v.1.0 and AllerTOP v.2.0 were unable to predict the allergenicity potential of Onc m 1.0101 (UniProt: P86431) and Onc m 1.0201 (Uniprot: P86432) due to the presence of multiple “X” residues in these sequences. These tools cannot process sequence with absent or nonstandard amino acids. Consequently, we considered these two sequences as non-allergen when calculating %sensitivity. Among the five *in silico* tools evaluated, AllerCatPro 2.0 achieved the highest sensitivity with 97.5%, followed by AlgPred 2.0 (93.7%), pLM4Alg-640 (89.9%), AllergenFP v.1.0 (88.6%) and AllerTop v.2.0 (83.5%) (Figure 3). AllerCatPro 2.0 correctly predicted allergenic potential with strong evidence for 77 of them, except for two shark PV isoforms, SPV-I (PDB: 5ZGM_A) and SPV-II (PDB: 5ZH6_A) from Spotless smooth-hound (*Mustelus griseus*), which were predicted with weak evidence. SPV entries were obtained from AllergenOnline and COMPARE and are based on IgE immunoblot analysis with fish-allergic patient sera in which stronger reactivity was attributed to SPV-I (Yang et al., 2018). The best-hit allergens for SPV-I and SPV-II were Gal d 8 (PVA from chicken) and Cro p 1 (PVB from Australian saltwater crocodile), respectively. Interestingly, a recent study showed that children with bony fish allergy tolerated Gummy shark (*Mustelus antarcticus*) (Dawes et al., 2025), a species belonging to the same genus as Spotless smooth-hound.

AlgPred 2.0 misclassified Pan h 9.0101 (pyruvate kinase M1/2b, RefSeq: XP_026775867.1), Pan h 11.01 (glucose-6-phosphate isomerase b, RefSeq: XP_026782721.2) and Sal s 9.01 (pyruvate kinase, UniProt: B5DGU1) as non-allergens. However, the Sal s 9 entry (https://allergen.org/viewallergen.php?aid=995) was no longer accessible as of January 2025. This is despite its inclusion as one of the 44 new food allergens added to the WHO/IUIS between January 2019 and March 2021. Notably, Sal s 9 was the only allergen among them that lacked detailed information (Sudharson et al., 2021).

For pLM4Alg-640, eight fish allergens were misclassified as non-allergens. Of these, seven were collagen α proteins: two from Barramundi (Lat c 6.0101 [RefSeq: XP_018521723.1] and Lat c 6.0201 [RefSeq: XP_018553992.1]), four from Atlantic Salmon (Sal s 6.0101 [RefSeq: XP_014059932.1], Sal s 6.0102 [RefSeq: XP_014048044.1], Sal s 6.0201 [RefSeq: XP_013998297.1], and Sal s 6.0202 ([RefSeq: XP_014033985.1]), and one from Rainbow trout (UniProt: BAB55663.1). Amino acid sequences exceeding 900 residues were excluded from the pLM4Alg study due to high computational demands. Consequently, these longer sequences, which exceeded 1,300 amino acids, were not recommended for prediction tasks using this tool (Du et al., 2024) and were misclassified. Additionally, the 333 amino acids long Pan h 10.01 (L-lactate dehydrogenase A chain, RefSeq: XP_026774991.1) was also misclassified as non-allergen from this tool.

For AllergenFP v.1.0, seven fish allergens were misclassified as probable non-allergens despite being listed in the WHO/IUIS database: Pan h 3.0101 (RefSeq: XP_026771637.1), Sal s 3.0101 (UniProt: B5DGM7), Pan h 7.01 (RefSeq: XP_026780620.2), Sal s 7.01 (GenBank: ACH70914.1), Sal s 8.01 (GenBank: ACM09737.1), Pan h 11.01(RefSeq: XP_026782721.2), Pan h 13.0101(RefSeq: XP_026782131.1). Similarly, AllerTOP v2.0 misclassified these seven allergens and four additional allergens as probable non-allergens: Pan h 9.0101 (RefSeq: XP_026775867.1), Sal s 9.01 (UniProt: B5DGU1), Pan h 8.01(RefSeq: XP_026795867.1) and Pan h 10.01 (RefSeq: XP_026774991.1). Table 3 ranks fish allergens based on their frequency of misclassification across the evaluated tools. Each allergen’s rank reflects the number of tools that misclassified it. For example, Pan h 11.01 was misclassified by three tools, making it the most frequently misclassified allergen, followed by Pan h 9.0101, Sal s 9.01 and Pan h 10.01, which were misclassified by two tools.

**TABLE 3:** Fish allergens most frequently misclassified by the allergenicity prediction tools.

Fish allergen	Biochemical name	Misclassifying tools	Number of tools
Pan h 11.01	Glucose 6-phosphate isomerase	AlgPred 2.0, AllergenFP v.1.0, AllerTOP v.2.0	3
Pan h 9.0101	Pyruvate kinase PKM-like	AlgPred 2.0, AllerTOP v.2.0	2
Sal s 9.01^a^	Pyruvate kinase	AlgPred 2.0, AllerTOP v.2.0	2
Pan h 10.01	L-lactate dehydrogenase	pLM4Alg-640, AllerTOP v.2.0	2

^a^ Sal s 9 entry (https://allergen.org/viewallergen.php?aid=995) from WHO/IUIS was no longer accessible as of January 2025.

In terms of best-hit allergens among AllerCatPro 2.0, AllergenFP v.1.0 and AllerTop v.2.0, only 49.4% (39/79) of the predicted allergens matched the input allergens in AllergenFP v.1.0 and AllerTop v.2.0. Conversely, 85% (67/79) of the predicted allergens matched the input allergens in AllerCatPro 2.0. In AllerCatPro 2.0, among the 77 allergens predicted with strong evidence of allergenicity, ten were identified as different allergens. Of these, six discrepancies arose because the best-hit allergen was an exact sequence match to the input allergen. However, these entries are not yet included in WHO/IUIS database but were obtained from AllergenOnline and COMPARE. For instance, PVB from European hake (UniProt: P02620) has 100% identity with the best-hit allergen PVB1 from Shallow-water Cape hake (Uniprot: P86756). Moreover, a limitation of AllerCatPro and other allergenicity prediction tools is that their known allergen datasets do not comprehensively include all fish isoallergens and their variants. This limitation sometimes results in queries being matched to protein isoforms from different fish species that are more similar to their own isoforms. For example, Onc m 1.0201 (Rainbow trout) was matched to Cyp c 1 (Common carp) as its best-hit allergen, since it had a higher %identity with Cyp c 1 (68.2%) compared to Onc m 1 (64.8%). Similarly, Pan h 4.0201 (Striped catfish) was best matched to Ore m 4 (Mozambique tilapia) rather than Pan h 4, due to its higher %identity with Ore m 4 (94.7% vs. 83.1). This issue highlights the need for prediction tools to expand their allergen databases to include all isoallergens and variants. Such enhancements could improve prediction accuracy and reduce discrepancies in matching results. To further account for potential bias from sequences already present in the allergen databases, we applied family-specific optimized thresholds. This strategy improves classification accuracy beyond simple sequence similarity and addresses isoform diversity and epitope-level allergenicity prediction.

### 3.3 Integrating B-cell epitopes information to optimize parvalbumin family-specific threshold using a phylogenetic tree

To improve allergenicity predictions using AllerCatPro 2.0, we incorporated experimentally validated epitope data for fish allergens from IEDB. Epitopes relevant to human allergic diseases were identified through both positive and negative assays. This search identified 93 B-cell epitopes from 11 fish species, all of which were linear except for one conformational epitope, based on 104 positive assay outcomes. Additionally, 83 B-cell epitopes from four fish species, all linear, were identified based on 118 negative assay outcomes (Table 4; full details are provided in Supplementary Table S2). In this study, we refer to epitopes from positive assay outcomes as “positive epitopes” and those from negative assay outcomes as “negative epitopes”.

**TABLE 4:** Fish allergen epitopes found in the Immune Epitope Database (IEDB).

No. Of B-cell epitopes	No. Of B-cell assays	No. Of fish species	Details
93	104 positive outcome assays	11	87 parvalbumins from 7 species: Atlantic cod; Atlantic salmon; Common carp; Atlantic mackerel; Hake (fragment); Grenadier; Silver hake 3 Tropomyosins: Yellow croaker 3 Collagens: Trout; Zebra fish; Flounder
83	118 negative outcome assays	4	82 parvalbumins from 3 species: Atlantic cod, Atlantic salmon, Common carp 1 Tropomyosins: Yellow croaker

Among the 93 positive epitopes, 87 were from PVs across seven fish species, three were from tropomyosin in yellow croaker, and the remaining three were from collagens in other species (Table 4). Of the 83 negative epitopes, 82 were from PVs in three species, and one was from tropomyosin in yellow croaker. Notably, yellow croaker was not included in the compiled database. No T-cell epitopes or MHC ligands were identified for fish allergens from IEDB.

**FIGURE 4:**
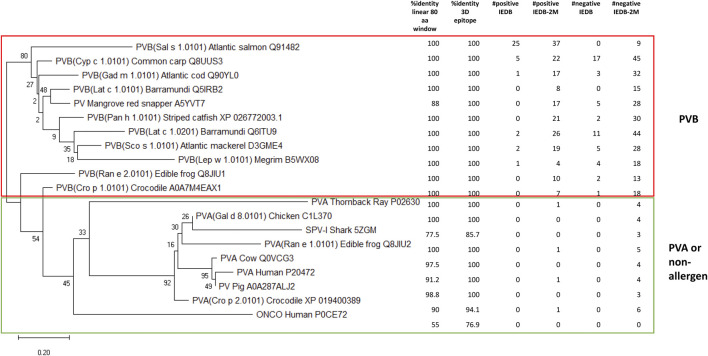
Phylogenetic tree for 20 representative parvalbumin (PV) isoforms. The first two columns show the AllerCatPro 2.0 results. The remaining four columns represent the number of positive IEDB epitopes mapped to the sequence (#positive IEDB), positive IEDB epitopes mapped with up to two mismatches allowed (#positive IEDB-2M), negative IEDB epitopes mapped to the sequence (#negative IEDB), and negative IEDB epitopes mapped with up to two mismatches allowed (#negative-2M). Eleven β -parvalbumins (PVB) are considered highly allergenic, while the remaining PVs, which include primarily α-parvalbumins (PVA), are considered low or non-.allergenic.

Next, a phylogenetic tree was constructed for PVBs, the major fish allergen, using representative PV sequences from the compiled fish dataset, along with PVs from other species, including red snapper, frogs, crocodiles, human, cow, pig, and chicken. Six key data points were mapped onto the tree: %identity in an 80-amino acid linear window, %identity for 3D epitope, #positive IEDB epitopes, #positive IEDB-2M (allowing up to two mismatches), #negative IEDB epitopes, and #negative IEDB-2M (refers to the Method section for details). As shown in Figure 4, 18 out of the 20 representative PV isoforms were predicted by AllerCatPro 2.0 as strong evidence (3Depi >93%). Only SPV-I from Spotless smooth-hound and human oncomodulin-1 were classified as weak evidence. Hence, using AllerCatPro 2.0 alone would not efficiently distinguish PVB from less allergenic proteins. On the other hand, by setting a family-specific optimization threshold of #positive IEDB-2M ≥ 4 effectively distinguishes PVB (a major fish allergen) from PVA (a less allergenic protein). This threshold also differentiates PVs from cartilaginous fish and non-allergen sources. Statistical analysis confirmed the distinction between PVB and PVA (or non-allergenic PVs) based on the number of matched epitopes per sequence, allowing up to two mismatches. Using a two-sample t-test with unequal variances (Welch’s t-test), PVBs had a significantly higher number of matched epitopes than PVAs or non-allergenic PVs (mean ± SD: 17.1 ± 9.6 vs. 0.44 ± 0.53; p = 0.00019, two-tailed), supporting the use of a family-specific threshold to improve prediction performance.

Among the 54 PVs in the compiled fish dataset, 96% (52 out of 54) had at least four #positive IEDB-2M epitopes, with only two exceptions. One of these was Pan h 1.0201 (RefSeq: XP_026803769.1), a WHO/IUIS-registered PV allergen from catfish, which notably had neither #positive IEDB epitopes nor #positive IEDB-2M (data not shown). In contrast, all other PV allergens registered in WHO/IUIS had at least one of these attributes, if not both. Pan h 1.0201 is an α-lineage PV that shares less than 67% amino acid identity with other PV allergens on WHO/IUIS (Dijkstra et al., 2024). It also has only 57% sequence identity with Pan h 1.0101. We previously reported that patients with IgE-binding to Pan h 1.0201 also reacted to Pan h 1.0101 and Sal s 1, suggesting some degree of cross-reactivity among these allergens despite the lower sequence identity (Ruethers et al., 2021). The second PV lacking both #positive IEDB epitopes and #positive IEDB-2M was SPV-I from Spotless smooth-hound (Figure 4). This finding is not surprising, as SPV-I belongs to the α-lineage PV, whereas SPV-II, which shares only 57% sequence identity with SPV-I, belongs to the β-lineage (Yang et al., 2018), which aligns with our result. SPV-II was found to contain nine #positive IEDB-2M epitopes, while SPV-I was identified as the closet evolutionary relative of the chicken PV allergen (Gal d 8.0101) (Figure 4). IgE immunoblot analysis showed that while sera from fish-allergic Chinese patients reacted to both SPV-I and SPV-II, the majority of sera exhibited stronger reactivity toward SPV-I than SPV-II (Yang et al., 2018). Notably, Pan h 1.0201 and SPV-I shared the highest pairwise identity (63%) among the 54 PVs in the dataset, whereas each exhibited <60% identity with the remaining 52 PV entries (data not shown). This finding highlights the complexity of establishing optimal thresholds for allergenicity predictions, which can be influenced by factors such as the availability, diversity and quality of epitope data. Additional patient IgE-binding data and clinical relevance information could further refine these thresholds. For instance, Mills et al. (Mills et al., 2024) introduced a ranking method for proteins based on their allergenic potential, using clinical relevance as a key criterion. For fish allergens, this study reviewed 39 papers, identified 45 allergenic sequences, and ranked them as High, Moderate, Low, or Very Low. Incorporating such attributes could enhance prediction accuracy. Additionally, Liu et al. reported that fish can be categorized into ‘PV-high’ and ‘PV-low’ groups based on allergen expression patterns, with higher PV expression correlating with elevated sIgE levels (Liu et al., 2024). While the predictive power was moderate, incorporating relative abundance data of PV isoforms from various fish species could further refine prediction accuracy. Overall, when considered collectively, our findings indicate that while the proposed threshold is promising and supported by both phylogenetic and epitope data, its applicability remains moderate and not yet fully generalizable, consistent with the limitations noted above.

## 4 Conclusion

In this study, we systematically compiled 79 ingested fish isoallergens and their variants from the four allergen databases, covering 34 fish species. Of these, 25 were common across all four databases. AllerCatPro 2.0 achieved the highest sensitivity (97.5%) in allergenicity prediction of fish allergens among the five *in silico* tools assessed. Our findings emphasize the need for these tools to expand their allergen datasets to include all isoallergens and their variants, which would enhance prediction accuracy and minimize discrepancies in matching results. Additionally, we proposed that by incorporating attributes such as the number of IEDB epitopes mapped with up to two mismatches (#positive IEDB-2M ≥ 4), we optimized a family-specific threshold to differentiate PVB, a major fish allergen, from PVA, a less allergenic protein. This threshold implementation will be useful for predicting whether putative allergenic PVs from fish species are likely to be more or less allergenic. However, further clinical data are needed to refine allergenicity predictions. The optimized thresholds have the potential to improve the performance of allergenicity prediction tools and can be applied to other protein families.

These findings highlight potential future applications. With additional patient IgE-binding data, clinical relevance information, and relative abundance data of PV isoforms, the optimized thresholds and comprehensive allergen datasets could inform diagnostics, support initial protein safety assessment, and help prioritize fish proteins for desensitization trials. They could also enable personalized management strategies based on the predicted allergenicity of specific isoallergens and variants. Further experimental studies are needed to evaluate these applications before practical implementation.

## Data Availability

The original contributions presented in the study are included in the article/Supplementary Material, further inquiries can be directed to the corresponding authors.
