# Supplementary figures and images for: Fish isoallergens and variants: database compilation, in silico allergenicity prediction challenges, and epitope-based threshold optimization
Source: Front Bioinform. 2025 Oct 20;5:1669237. doi: 10.3389/fbinf.2025.1669237 (PMC12580176; doi:10.3389/fbinf.2025.1669237)

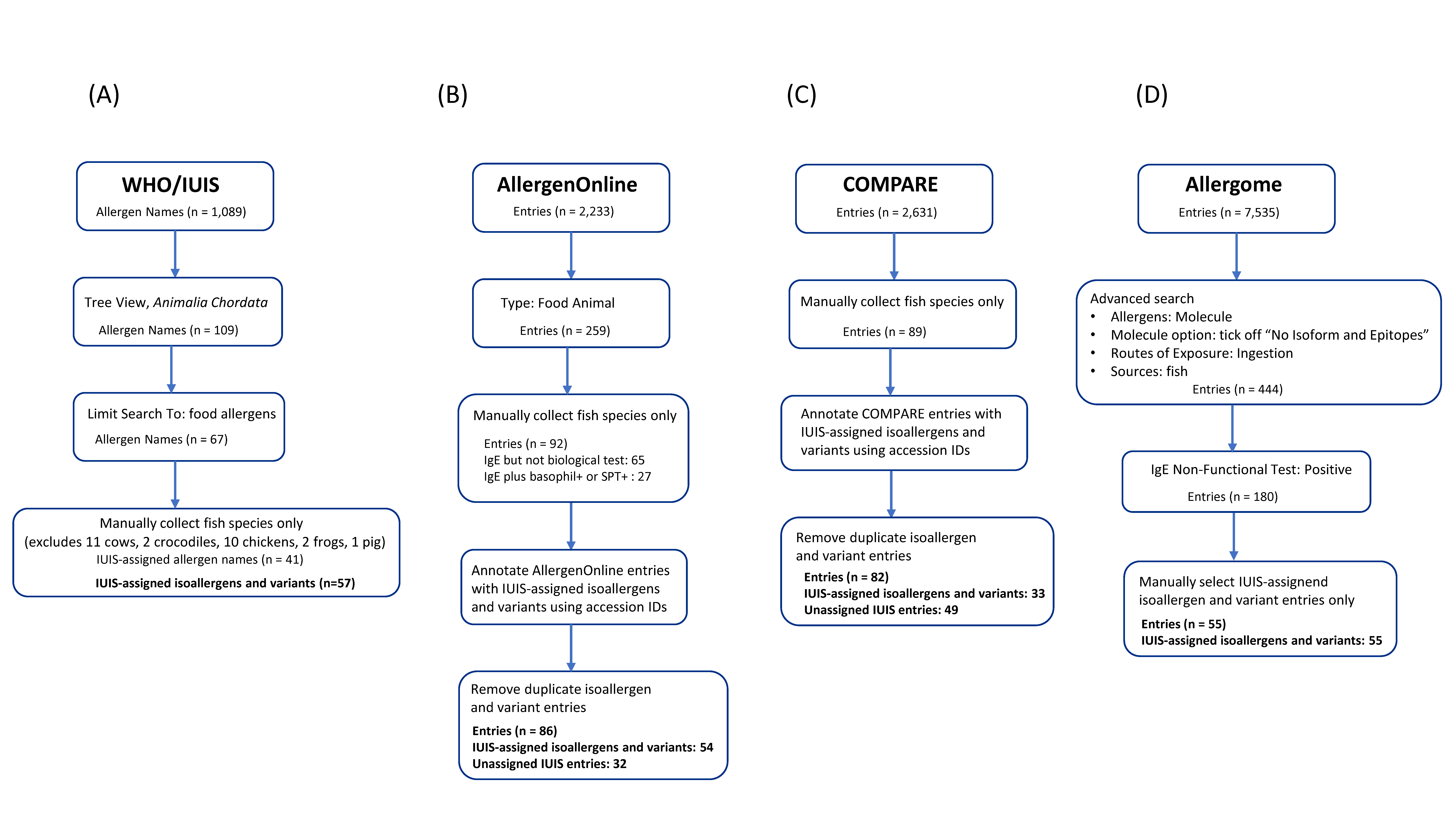

Supplement: Supplementary file 2 [file Image1.tif]
